# Intestinal evisceration and *Staphylococcus aureus* bacteremia due to ruptured umbilical hernia in a patient with liver cirrhosis: a case report and literature review

**DOI:** 10.1093/omcr/omac078

**Published:** 2022-07-26

**Authors:** Yuki Iizuka, Mayu Hikone, Yusuke Shimizu, Maki Tanabe, Kazuhiro Sugiyama, Yuichi Hamabe

**Affiliations:** Department of Emergency and Critical Care Medicine, Tokyo Bay Urayasu Ichikawa Medical Center, Chiba, Japan; Tertiary Emergency Medical Center (Trauma and Critical Center), Tokyo Metropolitan Bokutoh Hospital, Tokyo, Japan; Tertiary Emergency Medical Center (Trauma and Critical Center), Tokyo Metropolitan Bokutoh Hospital, Tokyo, Japan; Tertiary Emergency Medical Center (Trauma and Critical Center), Tokyo Metropolitan Bokutoh Hospital, Tokyo, Japan; Tertiary Emergency Medical Center (Trauma and Critical Center), Tokyo Metropolitan Bokutoh Hospital, Tokyo, Japan; Tertiary Emergency Medical Center (Trauma and Critical Center), Tokyo Metropolitan Bokutoh Hospital, Tokyo, Japan; Tertiary Emergency Medical Center (Trauma and Critical Center), Tokyo Metropolitan Bokutoh Hospital, Tokyo, Japan

## Abstract

Rupture of umbilical hernias is a potentially life-threatening condition that can occur in cirrhotic patients due to ascites. To the best of our knowledge, there are no previous reports on bacteremia following intestinal evisceration due to a ruptured umbilical hernia. Herein, we report a case of a 42-year-old female with a history of complicated alcoholic liver cirrhosis and schizophrenia who presented with intestinal evisceration and *Staphylococcus aureus* bacteremia secondary to a ruptured umbilical hernia. Due to a 2-day delay from presentation to hospitalization, the patient had a high risk for infection with skin flora. Initiation of appropriate antibiotic therapy, prompt surgical repair and adequate postoperative control of ascites markedly improved the patient’s condition. In cases of prolonged intestinal evisceration in adults with a ruptured umbilical hernia, bacteremia treatment with antibiotics coverage for skin flora should be considered.

## INTRODUCTION

In adults, umbilical hernias are caused by excessive abdominal wall distension due to ascites, obesity or intra-abdominal tumors. About 20% of patients with cirrhosis, especially those with massive ascites, develop umbilical hernias; however, rupture of umbilical hernias is extremely rare. Flood syndrome refers to the spontaneous rupture of an umbilical hernia with long-standing ascites [1], which may lead to sepsis and death [2]. Rarely, the omentum and intestines can eviscerate through the ruptured umbilical hernia, requiring urgent surgery.

Herein, we report a case of intestinal evisceration due to a ruptured umbilical hernia with *Staphylococcus aureus* bacteremia with liver cirrhosis and conduct a literature review of patient characteristics and treatment strategies of similar cases. To the best of our knowledge, this is the first report to describe a ruptured umbilical hernia with bacteremia.

## CASE REPORT

A 42-year-old female with a history of schizophrenia and complicated alcoholic liver cirrhosis (Child-Pugh class C) with gastroesophageal varices and ascites was transferred to our emergency and critical center for intestinal evisceration with ascitic fluid drainage from a ruptured umbilical hernia after straining during defecation 2 days prior.

**Figure 1:**
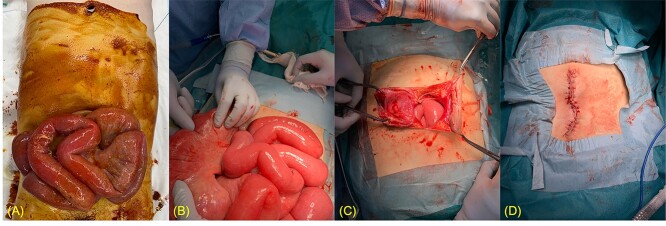
Eviscerated intestines due to ruptured umbilical hernia. (**A**) Mildly edematous small bowel eviscerated from the umbilical hernia. Povidone-iodine was used to clean the area. (**B**) A dilated and edematous segment of the small intestine measuring 130–230 cm from the ligament of Treitz. (**C**, **D**) Surgical repair.

**Figure 2:**
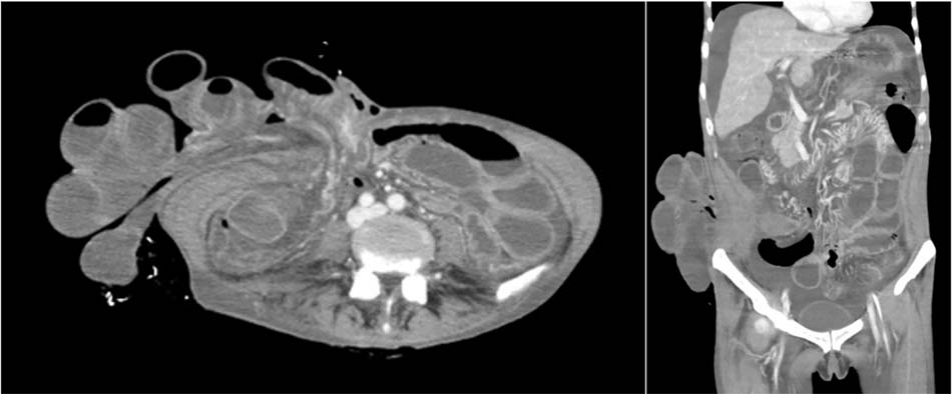
Abdominal computed tomography showing liver atrophy and intestinal evisceration due to ruptured umbilical hernia.

Assessment of vital signs on admission showed tachycardia (122 bpm), tachypnea (24 bpm) and hypotension (96/67 mmHg) with normal mental status (Glasgow Coma Scale score: E4V5M6), body temperature (35.5°C) and oxygen saturation (98% in room air). Physical examination revealed evisceration of mildly edematous small bowel from the umbilicus and persistent ascitic fluid drainage with no signs of ischemia (Fig. 1**A**). Abdominal computed tomography revealed intestinal evisceration from the umbilical region (Fig. 2).

After resuscitation with a crystalloid fluid bolus and vasopressor support, urgent surgical repair was performed. A 5-cm skin incision was made above and below the umbilicus to open the abdomen. Intraoperatively, a dilated and edematous segment of the small intestine measuring 130–230 cm from the ligament of Treitz was eviscerated through the ruptured umbilical hernia (Fig. 1**B**) with a serosal tear repaired using 3–0 Vicryl. Regardless, the intestines were easily reduced into the abdomen. There were no signs of ischemia that required bowel resection. The umbilicus was trimmed and primarily closed without mesh placement due to the infection risk (Fig. 1**C** and **D**).

Antibiotic therapy with ampicillin/sulbactam and vancomycin was empirically initiated on the day of arrival to target both skin and colonic flora. Blood cultures obtained on the first day of hospitalization were positive for methicillin-sensitive *S. aureus*. Fortunately, echocardiography ruled out endocarditis. These findings indicated that intestinal evisceration was the only etiology of the bacteremia. Follow-up blood cultures were negative, which prompted an antibiotic switch to cefazoline. The patient was extubated and weaned off from vasopressor support on the third and sixth hospital days, respectively.

Although her clinical course was complicated by bacterial peritonitis and refractory ascites requiring repeated therapeutic paracenteses, these were eventually controlled with combined diuretic therapy (i.e. furosemide, spironolactone and tolvaptan). She was discharged from the intensive care unit and was sent home on the 16th and 23rd hospital days, respectively.

## DISCUSSION

Ruptured umbilical hernias are commonly caused by local skin trauma, vomiting and endoscopy due to increased intra-abdominal pressure. Skin color changes at the umbilical hernia site, ulcerations and rapid increase in size may be suggestive of rupture [2]. Ruptured umbilical hernias may be triggered by straining during defecation, similar to our case [3].

We searched literature dating from 1990 on adults with liver cirrhosis who had a ruptured umbilical hernia on PubMed using ‘umbilical hernia’ and ‘evisceration’ as keywords. From a total of 27 articles found, only six articles reporting seven cases matched the topic of this study [3–8].

The patients’ characteristics, treatment (antibiotic and surgical) and outcomes are summarized in Table 1. Most of these patients were male ≥40 years old. Alcoholic liver disease was the most common cause of liver cirrhosis. Among the eight patients (including ours), only four survived. Moreover, none of these previously reported cases involved bacteremia, unlike our case.

**Table 1:** Clinical review of reported cases of intestinal or omental evisceration due to ruptured umbilical hernia in adult patients with liver cirrhosis

Case No.	Author	Age	Sex	Etiology of cirrhosis	Child-Pugh	Evisceration	Antibiotics	Surgical treatment	Ascites	Outcome
1	Arora et al. [3]	42	F	Hepatitis C	C	Omentum	−	Urgent surgery; partial omentectomy with primary repair	−	Death
2	Arora et al. [3]	40	M	Alcohol	C	Omentum	3rd cephalosporin	Elective surgery; primary repair	PVS	Death
3	Ginsburg et al. [4]	40	M	Alcohol	−	Omentum	Cefotetan	Elective surgery; partial omentectomy with primary repair	Paracentesis, diuretics, PVS	Death
4	Choo et al. [5]	41	M	Alcohol	−	Small bowel	ABPC/SBT	Urgent surgery; primary repair	Diuretics	Survived
5	Good et al. [6]	81	M	Alcohol	−	Omentum	−	Urgent surgery; partial omentectomy with primary repair	−	−
6	Ogu et al. [7]	50	M	Hepatitis C, alcohol	−	Small bowel	−	Urgent surgery; primary repair	−	Survived
7	Albeladi et al. [8]	23	F	Hepatitis B	B	Omentum	Broad-spectrum	Urgent surgery; primary repair	Drain placement	Survived
8	Our case	42	F	Alcohol	C	Small bowel	ABPC/SBT, VCM	Urgent surgery; primary repair	Paracentesis, diuretics	Survived

M, male; F, female; PVS, peritoneovenous shunting; ABPC/SBT, ampicillin/sulbactam; VCM, vancomycin.

The choice between conservative management and surgical repair for ruptured umbilical hernias remains controversial [9]. However, from our literature review, most cases with omental or intestinal evisceration required urgent surgical mesh repair, and primary closure with suturing was preferred due to the risk of infection [3, 5, 9] as in our case.

In these reported cases, antibiotics were administered prophylactically or as treatment for peritonitis in addition to urgent surgery. Although there is no fixed evidence or guideline for selecting the appropriate antibiotics, previous reports suggest using third-generation cephalosporins and carbapenems to cover organisms typical of bacterial peritonitis. Our case highlights the importance of antibiotic therapy to cover skin and colonic flora since *S. aureus* bacteremia could occur due to a ruptured umbilical hernia, especially when the intestine is eviscerated and exposed for longer periods. It has been shown that patients with cirrhosis tend to have bacteremia due to increased intestinal membrane permeability. Furthermore, the bacteremia risk may vary depending on the severity of cirrhosis and the duration of intestinal evisceration [10].

Since a ruptured umbilical hernia in cirrhotic patients is a consequence of increased intra-abdominal pressure due to refractory ascites, controlling ascites is important in postoperative management to prevent a recurrence. Although using diuretics alone may not be a promising strategy, repeated therapeutic paracenteses may be necessary in the acute postoperative phase or when complicated with infection. Combining short-term drain placement [8], transjugular intrahepatic portocaval shunt placement [11] and peritoneovenous shunts [3, 4] have been described previously. However, their utility is generally limited to specific indications in selected patients.

The distinctive factor of our case was a 2-day delay from the rupture of the umbilical hernia to the hospital visit, allowing bacteremia to develop. This highlights the significance of urgent medical therapy with antibiotics covering the skin flora and surgery in patients with intestinal evisceration secondary to a ruptured umbilical hernia.

In conclusion, rupture of the umbilical hernia with intestinal evisceration in cirrhotic patients is a rare but potentially life-threatening disease that often warrants intensive care, including adequate antibiotic selection and management of ascites after surgery. This case illustrates the importance of selecting appropriate antibiotics against skin flora to reduce the risk of bacteremia in patients with eviscerated intestines following the rupture of umbilical hernia.
